# A Comparative Analysis of Differences in Salivary hBD-2 Levels and Their Correlation with Dental Caries and Unstimulated Saliva pH in Children with Primary and Permanent Dentition

**DOI:** 10.3390/diagnostics16040591

**Published:** 2026-02-16

**Authors:** Branislava Stojković, Marija Igić, Tatjana Jevtović Stoimenov, Olivera Tričković Janjić, Aleksandra Ignjatović, Miloš Kostić, Milica Petrović, Simona Stojanović, Nikola Živković, Ana Stojanović, Zorana Veličković

**Affiliations:** 1 Department of Dentistry, Medical Faculty Niš, University of Niš, 18 000 Niš, Serbia; marija.igic@medfak.ni.ac.rs (M.I.); olivera.trickovic.janjic@medfak.ni.ac.rs (O.T.J.); milica.petrovic@medfak.ni.ac.rs (M.P.); simona.stojanovic@medfak.ni.ac.rs (S.S.); ana.stojanovic@medfak.ni.ac.rs (A.S.); zorana.velickovic@medfak.ni.ac.rs (Z.V.); 2 Department of Biochemistry, Medical Faculty Niš, University of Niš, 18 000 Niš, Serbia; tatjana.jevtovic.stoimenov@medfak.ni.ac.rs; 3 Department of Medical Statistics and Informatics, Medical Faculty Niš, University of Niš, 18 000 Niš, Serbia; aleksandra.ignjatovic@medfak.ni.ac.rs; 4 Department of Immunology, Medical Faculty Niš, University of Niš, 18 000 Niš, Serbia; milos.kostic@medfak.ni.ac.rs; 5 Department of Pathology, Medical Faculty Niš, University of Niš, 18 000 Niš, Serbia; nikola.zivkovic@medfak.ni.ac.rs

**Keywords:** saliva, human beta-defensin 2, dental caries, salivary pH, children

## Abstract

**Background/Objectives**: This exploratory study aimed to investigate potential differences and associations between salivary hBD-2 levels and dental caries, as well as between unstimulated salivary pH and salivary hBD-2 levels, in children with primary and permanent dentition, aiming to profile these two dentition-development groups. **Materials and Methods**: This cross-sectional study included children with primary (*n* = 75) and permanent dentition (*n* = 78). Data were collected by surveying mothers and clinically exam ining their children. Unstimulated saliva samples of the children were collected to determine salivary pH and hBD-2 levels. Spearman’s rank correlation coefficient was used to assess the correlation between the examined variables. **Results**: Children with permanent dentition had a significantly higher mean salivary hBD-2 level (*p* = 0.044), while children with primary dentition had a significantly higher mean unstimulated saliva pH (*p* < 0.001). No significant correlation was determined between salivary hBD-2 levels and dental caries, nor between unstimulated saliva pH and dental caries in either group of study participants (hBD-2: *p* = 0.515 and 0.224; pH: *p* = 0.121 and 0.061, respectively). A significant negative association between salivary hBD-2 peptide levels and unstimulated saliva pH in children with permanent dentition was revealed (*r_s_* = −0.230, *p* = 0.043). **Conclusions**: Salivary hBD-2 levels were higher in children with permanent dentition, with an inverse association with unstimulated saliva pH observed only in this group. No significant correlation was found between salivary hBD-2 levels and dental caries. Further well-designed studies are needed to better understand dentition or age-related variations in salivary hBD-2 levels, their association with unstimulated salivary pH, and their potential as a caries biomarker.

## 1. Introduction

Non-immunoglobulin antimicrobial proteins in saliva are integral components of the innate immunity of the oral cavity. They represent the first line of defense against invasion by various pathogens and are therefore of particular importance for maintaining oral homeostasis. These proteins are classified into six functional groups and ranked from small cationic peptides to large agglutinating proteins, with each functional group having a specific mechanism of antimicrobial action [1,2].

In recent decades, extensive research has clarified the roles of salivary cationic antimicrobial peptides in maintaining oral homeostasis and microecosystem integrity. These are small peptides composed of 12 to 50 amino acids, which, at physiological pH values, carry a positive charge originating from the excess of basic residues (arginine, lysine, histidine) compared to acidic amino acid residues [3,4,5]. Defensins and cathelicidins are among the most studied cationic antimicrobial peptides due to their biological significance [1,5,6,7,8,9,10]. In human oral biology, defensins are of particular interest because of their potent antimicrobial properties in the oral environment. They are small, low-molecular, cationic peptides characterized by the presence of six to eight cysteine residues linked by three to four intramolecular disulfide bonds [11,12]. In humans, two subfamilies of defensins have been identified: α-defensins and β-defensins. β-defensins play a critical role in maintaining oral homeostasis and regulating various oral diseases [13,14,15,16,17]. Within the human oral cavity, four β-defensins—designated human β-defensin 1, 2, 3, and 4 (hBD-1, -2, -3, -4)—have been characterized. The genes encoding β-defensins are located on the chromosomal region 8p21–p23 [18].

Although all human β-defensins are crucial for the maintenance of oral homeostasis, human β-defensin 2 (hBD-2), a cationic peptide composed of 41 amino acids, has attracted considerable scientific attention. In the oral cavity, this peptide is produced by epithelial and salivary gland cells, although fibroblasts and monocytes have also been shown to contribute to its production [19,20]. Like other cationic antimicrobial peptides, they are characterized by a three-dimensional amphiphilic structure [21]. Despite having different antimicrobial properties, their activity is primarily directed against Gram-negative (G-) oral bacteria, including cariogenic microorganisms, which they kill rapidly, acting as strong bactericidal and/or bacteriostatic agents [22,23,24,25]. However, the mechanism of antibacterial action of hBD-2 is quite complex. This peptide exerts its antibacterial activity through direct effects on bacteria via both extracellular (through strong electrostatic interaction between hBD-2 peptides and the bacterial envelope; following the initial interaction, peptides bind to the bacterial membrane surface, aggregate on the membrane, and form channels and transmembrane pores, resulting in osmotic lysis and cell death) and intracellular mechanisms (by inhibiting intracellular processes within bacteria, primarily through the inhibition of enzymatic activity and the synthesis of the cell wall, DNA, RNA, and proteins) [24,26,27]. Cationic hBD-2 also affects the acquired immune response by stimulating the production of proinflammatory cytokines [28,29,30,31,32]. Moreover, hBD-2 also plays both anti-inflammatory and anti-cancer roles, as well as a role in post-extraction wound healing [4].

Gene expression and secretion of this peptide are induced by various agents [3]. These agents first stimulate the release of proinflammatory cytokines, including IL-1, TNF-α, and IFN-γ, which then stimulate hBD-2 expression [3,33,34] by the mitogen-activated protein kinase (MAPK) pathway and nuclear factor kappa B (NFκB) activation [34,35]. The promoter region of the hBD-2 gene has been shown to contain binding sites for both activator protein-1 (AP-1), a transcription factor activated by the MAPK pathway, and NF-κB [36]. First, hBD-2 is synthesized as a preprodefensin that is proteolytically processed into a cationic mature peptide and subsequently secreted into oral fluids, including saliva and gingival crevicular fluid [6].

Salivary secretion of this peptide is largely induced by antimicrobial and inflammatory agents, as well as oral tissue injuries [3,37,38]. Oral microorganisms also participate in the stimulation of salivary secretion of this peptide, which is confirmed by the positive correlation of salivary hBD-2 levels with various oral microorganisms [21]. Furthermore, the level of hBD-2 in saliva can also be affected by numerous systemic factors such as hyperglycemia and long-term use of supplements based on vitamins A and D. Some studies have also suggested that the level of hBD-2 in saliva changes with age [20]. Nevertheless, most of these studies focused on the adult population, and to date, there are no data in the available literature regarding age-related changes in the salivary level of this peptide in children [39,40]. Recently, it has been pointed out that the salivary level of the cationic antimicrobial peptide cathelicidin LL-37 increases with the age of children, whereas such data are lacking for defensins, including hBD-2 [41]. Furthermore, studies have shown that certain antimicrobial proteins reach adult values already in early childhood, but similar data for hBD-2 are still unavailable [42].

Since many oral microorganisms are highly sensitive to hBD-2, this peptide is considered both an important natural defense factor and a potential biomarker and therapeutic agent for various oral diseases [19,43,44,45,46,47]. From the pediatric dental perspective, the role of hBD-2 in the biological control of caries is particularly important, as well as its potential role as a caries biomarker. Biomarkers are defined as quantitative biological parameters that represent indicators of a pathophysiological condition, or for which studies have established a relationship with disease [48]. However, the results from the literature regarding the correlation between salivary hBD-2 level and dental caries in children are contradictory [49,50,51,52,53,54,55,56].

However, in dynamic environments such as saliva, the concentration of active forms of hBD-2 may be conditioned by different factors, including different ions and the pH of the environment [57,58,59]. Although it might be expected that salivary hBD-2 correlates with salivary pH, there are no precise data in the available literature to support this. Such data could be important because pathological processes in the oral cavity generally occur in an acidic environment, and would also contribute to a better understanding of the biology and role of this peptide in the oral environment in children. Regarding the oral environment, the analysis of the correlation between the salivary level of this peptide and the pH of unstimulated saliva is particularly significant, given its longer presence in the oral cavity compared to stimulated saliva.

Considering all the above, an exploratory study was conducted to investigate potential differences and associations between salivary hBD-2 levels and dental caries, as well as between unstimulated salivary pH and salivary hBD-2 levels, in children with primary and permanent dentition, aiming to profile these two dentition-development groups.

## 2. Materials and Methods

### 2.1. Study Design and Participants

Using a cross-sectional analysis, this study analyzed children with primary and permanent dentition who were patients of the Preventive and Pediatric Dentistry Department of the Clinic of Dental Medicine at the Faculty of Medicine, University of Niš in Serbia, and patients of the Department of Preventive and Pediatric Dentistry of the Health Centre in Niš from 3 kindergartens and 3 elementary schools within the central area of the City of Niš. Upon providing detailed verbal and written information about the study objectives and methodology, as well as measures taken to protect the identity of the participants, the directors of these institutions gave written consent for participating in this study. Additionally, the mothers of the participants were thoroughly acquainted with the methods and aims of this study, as well as the protection of their children’s identity. This information was initially communicated verbally through a formal conversation, followed by written informed consent, both of which featured completely the same content. Mothers of study participants had an unrestricted opportunity to request further elucidation or more detailed clarification to ensure a precise, comprehensive, and informed understanding of all components of this study. By signing it, all of the mothers confirmed this informed consent statement (which was uniform for all mothers of the participants), thus allowing their children to participate in this study. Finally, the performance of this study in the mentioned institutions was approved by the Ethics Committee of the Faculty of Medicine, University of Niš, Serbia (Decision No. 12-14532-2/3), and the study itself was carried out in compliance with all the principles of the Declaration of Helsinki.

### 2.2. Inclusion and Exclusion Criteria

Inclusion criteria were as follows: children (1) with complete primary dentition and children with complete permanent dentition; (2) born in the City of Niš, Serbia, and with permanent residence in the city since birth (the average fluoride concentration in drinking water is <0.05 mg/mL [60]); (3) with no history of systemic diseases and no acute infectious diseases at the time of examination; (4) not receiving any medication or vitamin supplementation; (5) who had not used antibiotics for at least 30 days prior to the onset of the study; (6) without diseases and injuries of soft oral tissues; (7) with all healthy teeth or with healthy teeth and teeth with untreated caries (decayed teeth—dt/DT); (8) with whom cooperation could be reliably established during the clinical examination and saliva collection by spitting into sterile test tubes, and who demonstrate visually adequate salivary flow; (9) whose mothers provided signed informed consent statement for the participation of their children in this study.

Exclusion criteria were as follows: children with (1) filled and extracted teeth; (2) structural defects on the teeth; (3) traumatic injuries of teeth and soft tissues; (4) poor or absent oral hygiene (OHI-S ≥ 1); (5) a gingival index ≥ 1; (6) oral soft tissue diseases.

Study participants were selected based on medical/dental records, school registries, clinical examination, and information obtained by surveying their mothers.

### 2.3. Data Collection

Data were collected between March and May 2017 in dental offices of the institutions included in this study (each participating kindergarten and elementary school had an organized dental service and its own dental office, which is part of the Department of Preventive and Pediatric Dentistry at the Health Centre in Niš, Serbia). The mothers of the children, as well as the children themselves, i.e., children with permanent dentition, received all instructions necessary as prerequisites for the data collection. Data were collected during a single visit, in the morning hours, approximately between 9:00 AM (CET) and 10:00 AM (CET), i.e., one hour after the usual oral routine, on an empty stomach. The data were collected by a well-trained examiner with many years of experience as a specialist in preventive and pediatric dentistry.

#### 2.3.1. Basic Data Collection

Basic demographic data of the study participants, data on their health status, potential medication and supplement use, and antibiotic use within 30 days prior to this study were obtained by surveying the mothers.

#### 2.3.2. Clinical Examination of the Study Participants

The clinical dental examination of the study participants was performed in a dental chair under artificial lighting using a dental mirror, a standard dental probe, and a probe with a rounded tip (Goldman-Fox, Hu-Friedy, Mfg Co., Inc., Chicago, IL, USA).

The health status of soft oral tissues was assessed using a standard/conventional examination of the oral cavity, in accordance with WHO recommendations [61]. The modified DDE index [62] was used to evaluate structural defects. The state of oral hygiene in children with permanent dentition was assessed using the Simplified Oral Hygiene Index (OHI-S) [63], and its modification according to Thwin et al. in children with primary dentition, where vestibular surfaces of teeth 55, 51, 65, and 71 and oral surfaces of teeth 75 and 85 were selected as reference teeth for oral hygiene assessment [64]. Gingival health was assessed using the Löe–Silness index [65]. Caries diagnosis was performed following the World Health Organisation (WHO) criteria for epidemiological studies in order to determine the decayed, missing, and filled teeth index (dmft/DMFT), i.e., the decayed teeth index (dt/DT index) [61].

#### 2.3.3. Unstimulated Saliva Sampling and Measurement of pH and hBD-2 Antimicrobial Peptide Levels

Immediately upon dental examination, unstimulated saliva samples were collected from the study participants through spitting into sterile test tubes. The pH value of unstimulated saliva was measured in the dental office after sampling (about 1 mL) using a digital, portable pH meter (Hanna Instruments, Woonsocket, RI, USA). Calibration of the pH meter and pH measurement of unstimulated saliva were carried out in accordance with the manufacturer’s recommendations. After determining the pH of the unstimulated saliva, it was sampled in an identical manner to determine the level of hBD-2 antimicrobial peptide in it. The saliva was collected through spitting into sterile tubes over 5–10 min, to obtain 2 mL of unstimulated saliva. The samples were stored at +2 °C and within a maximum of one hour after sampling were transported to the Scientific Research Centre for Biomedicine, Faculty of Medicine, University of Niš, Serbia, for further analysis. The samples were first centrifuged at 10,000 rpm at +4 °C for 10 min. The resulting supernatant was separated, and the samples were frozen at −82 °C until the next phase. Salivary concentrations of the antimicrobial peptide human β-defensin 2 (hBD-2) were determined using a sandwich enzyme-linked immunosorbent assay (ELISA). Measurements were performed with a commercial ELISA kit (Cusabio Human β-Defensin 2 ELISA Kit; CSB-E13201h; lot no. P18060867, Wuhan, China), in accordance with the manufacturer’s instructions. According to the manufacturer’s specifications, the assay detection range was 62.5–4000 pg/mL, with a sensitivity (lower limit of detection) of 15.6 pg/mL. The reported intra-assay and inter-assay coefficients of variation were <8% and <10%, respectively. For consistency with the available literature, hBD-2 concentrations were expressed in ng/mL.

### 2.4. Sample Size

The sample size in this study was determined based on a priori power analysis for detecting a medium effect size (d) in the difference in hBD-2 in participants with primary dentition and permanent dentition. With an alpha level of 0.05 (two-tailed test) and a power of 0.80, the representative sample size was calculated to be 128 participants. Sample size calculation was performed in G*Power 3.1.9.7.

### 2.5. Statistical Analysis

The “R” software package (version 4.5.1; R Foundation for Statistical Computing, Vienna, Austria) was used for statistical analysis of the obtained data [66]. Data are presented in the tables and graphs in the form of arithmetic mean, standard deviation, and median, minimum, and maximum values, i.e., in the form of absolute and relative numbers. Data distribution was assessed using the Shapiro–Wilk test. Normally distributed data were analyzed using the Student’s t-test, and non-normally distributed data were analyzed using the Mann–Whitney test. The association between the examined independent variables was evaluated using Spearman’s rank correlation coefficient, with a 95% confidence interval. Effect size was interpreted according to Cohen’s guidelines for correlation coefficients: negligible (<0.1), small (0.1–0.3), medium (0.3–0.5), and large (>0.5). The null hypothesis was tested using a significance threshold of *p* < 0.05.

## 3. Results

A total of 269 children were examined, of whom 75 with complete primary dentition met the inclusion criteria and were included in the primary dentition group. A total of 247 children were examined, 78 children with permanent dentition met the inclusion criteria and were included in the permanent dentition group. Therefore, this study included 153 participants. Basic demographic and clinical characteristics of the study participants are shown in Table 1.

**Figure 1:**
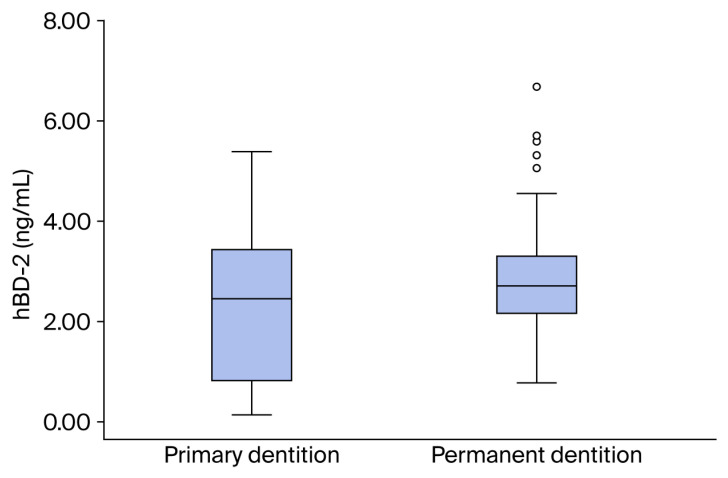
Salivary hBD-2 values in both groups of study participants.

The examined hBD-2 peptide was detected in unstimulated saliva samples of all 153 study participants, with its mean level being significantly higher in children with permanent dentition compared to children with primary dentition (*p* = 0.044). Mean values of unstimulated saliva pH are significantly higher in children with primary dentition compared to children with permanent dentition (*p* < 0.001) (Table 2, Figure 1).

In both groups of study participants, hBD-2 peptide levels were higher in children with untreated caries compared to caries-free children; however, the differences were not statistically significant (*p* = 0.535 and *p* = 150, respectively). Similarly, the pH values of unstimulated saliva were lower in children with untreated caries across both groups of study participants, but without statistically significant differences in comparison to the caries-free participants, as shown in Table 3.

No significant correlation was determined between salivary hBD-2 concentration and dental caries (*p* = 0.515 and 0.224, respectively), nor between unstimulated saliva pH and dental caries (*p* = 0.121 and *p* = 0.061, respectively) in either group of study participants (Table 4). Correlation analysis revealed a significant negative association between salivary hBD-2 peptide levels and unstimulated saliva pH in children with permanent dentition (*r_s_* = −0.230, *p* = 0.043) (Table 4). This correlation was not found in children with primary dentition (*r_s_* = −0.067, *p* = 0.566) (Table 4).

## 4. Discussion

In this study, the relationship of salivary hBD-2 levels with dental caries and unstimulated saliva pH in children with primary and permanent dentition was analyzed. This study found no significant correlation between salivary hBD-2 levels and dental caries in either children with primary dentition or those with permanent dentition. However, it was also found that mean salivary hBD-2 levels were higher in children with permanent dentition compared to those with primary dentition. A significant negative correlation between salivary hBD-2 levels and unstimulated saliva pH was observed in children with permanent dentition, while no such correlation was found in children with primary dentition.

This study included children with primary dentition, with a mean age of 3.44 years, and children with permanent dentition, with a mean age of 12.63 years. It did not include children with mixed dentition due to the heterogeneity of age and dentition, which limits accurate assessment of caries prevalence, and because mixed dentition may act as a confounding factor, primarily of the salivary level of the investigated peptide.

As noted, previous findings regarding the correlation between salivary hBD-2 peptide levels and dental caries have been inconsistent. In the present study, this correlation was not observed. These results align with those reported by Phattarataratip et al. [51], Ribeiro et al. [52], and Nazemisalman et al. [53]. The discrepancies among various studies may stem from differences in caries diagnostic criteria or variations in sample sizes. To ensure data robustness, we applied strict inclusion criteria (e.g., systemically healthy children, no recent medication, low OHI-S and gingival index scores); however, salivary hBD-2 levels remained independent of the caries status in our cohort. While dental caries is a multifactorial disease driven by microbial and environmental factors, our findings suggest that at this exploratory level, hBD-2 concentration does not serve as a direct marker for caries experience in the studied population. This lack of association underscores the complexity of the innate immune response in the oral cavity, which warrants further longitudinal investigation. Once these microorganisms colonize the oral cavity, proinflammatory cytokines are released, which stimulate the expression of hBD-2 peptide in saliva [67,68,69]. Studies have also established a significant association of salivary hBD-2 with cariogenic microorganisms, primarily *S. mutans* [70]. Consequently, some authors suggest that salivary hBD-2 peptide levels correlate more strongly with the presence of cariogenic microorganisms in saliva than with caries lesions, which represent the final stage of the caries process [71]. On the other hand, salivary hBD-2 activity is not highly specific to a single microorganism or pathogen. Therefore, assessing its association with dental caries in children requires careful participant selection. Although the present study sought to exclude conditions for which there is evidence that they may influence salivary hBD-2 levels, as well as certain dental confounding factors, the examined correlation was not observed. As previously noted, hBD-2 exhibits a complex mechanism of action, encompassing both direct antimicrobial activity and modulation of adaptive immunity, acting independently or synergistically with other antimicrobial proteins. Therefore, to clarify the role of hBD-2 peptides as a caries biomarker—given the multifactorial etiology of dental caries—further studies with carefully selected participants and consideration of additional potential confounding factors are needed. Additionally, given the complex antimicrobial and immunomodulatory functions of hBD-2, a multi-marker approach should be considered.

The values of hBD-2 levels in unstimulated saliva registered in this study are generally consistent with those reported in children by other authors, showing individual variations that were more pronounced in children with primary dentition [49,50,51]. Also, the present study found that mean salivary hBD-2 levels were higher in children with permanent dentition compared to those with primary dentition. Similar observations have been reported for human cathelicidin LL-37, another cationic peptide, by Davidopoulou et al. [41]. However, since there are no similar results for salivary hBD-2 levels in children in the available literature, direct comparison was not possible. Still, in vitro studies have shown that salivary hBD-2 levels increase with age [72], which supports the results obtained in this study. The higher salivary hBD-2 levels observed in children with permanent dentition, compared with those with primary dentition, may be attributed to differences in salivary physiology, oral microbiota composition, and immune system maturation. It is likely that this positive correlation is based on continuous changes in oral microflora during childhood, which constantly stimulate the effector mechanisms of innate immunity of the oral cavity, causing an age-related increase in salivary peptide levels [73]. This applies primarily to cationic peptides originating from oral epithelial cells, such as hBD-2. On the other hand, Shimizu et al. found that salivary hBD-2 levels in individuals older than 65 are lower than in younger adults aged 22–30 [39]. This result could be explained by age-related changes in the composition of oral flora (e.g., loss of *S. mutans* with toothlessness), as well as by age-related atrophy of the oral epithelium. Hence, the results of the current study, consistent with previous studies, underscore the need for rigorously designed research to clarify the age-related physiological dynamics of hBD-2 secretion from childhood through adulthood.

Finally, a significant negative correlation between salivary hBD-2 levels and unstimulated saliva pH was observed in children with permanent dentition. No such correlation was detected in children with primary dentition, which may be partly due to the greater variability in peptide levels and the higher unstimulated saliva pH observed in this group. Since there are no similar studies in the available literature, the obtained results cannot be compared. However, it is supported by Bachrach et al., who found that one of the cationic peptides, cathelicidin LL-37, was not detected in parotid saliva, whose pH is significantly higher than that of unstimulated saliva [74]. An inverse relationship was observed between salivary hBD-2 levels and the pH of unstimulated saliva in children with permanent dentition, suggesting that the concentration of active forms of this peptide is higher in acidic environments. This finding is particularly relevant, as pathological processes in the oral cavity, including dental caries, often occur under such conditions. Evidence indicates that hBD-2 contributes to preventing dental biofilm formation, and studies have shown that the levels of host antimicrobial peptides can influence the colonization of specific cariogenic microorganisms, including S. mutans [44,75]. While our findings show an inverse correlation exclusively in children with permanent dentition, the limited effect size and cross-sectional nature of this study preclude definitive conclusions. Further research is needed to clarify the physiological dynamics and potential clinical implications of these associations.

This study has several notable limitations. The main limitations of this study are the small sample size and limited age range, which restrict the assessment of age-related changes in salivary hBD-2 levels. In addition, the relationship between hBD-2 and unstimulated salivary pH remains unclear. Furthermore, caries assessment using WHO criteria may be less sensitive than alternative approaches such as ICDAS [76]. Although participants were carefully selected to minimize potential confounding factors, stratified analyses or statistical adjustments for variables such as gender, dietary habits, oral hygiene practices, and socio-economic factors were not performed. Additionally, observed associations between hBD-2 and clinical parameters should be interpreted as exploratory, as examining multiple correlations without formal correction might increase the risk of Type I error. Future well-designed studies with larger cohorts are needed to better define the role of salivary hBD-2 as a caries biomarker, explore its association with salivary pH, and clarify age-related changes.

## 5. Conclusions

This exploratory study found higher salivary hBD-2 levels in children with permanent dentition, with an inverse association with unstimulated saliva pH observed only in this group. However, this study has not found a significant correlation between salivary hBD-2 levels and dental caries in children. Well-designed studies with carefully selected participants are required to delineate dentition, i.e., age-related differences in salivary hBD-2 levels, and to systematically assess associations between salivary hBD-2, dental caries, and unstimulated salivary pH, thereby advancing current understanding of the role of hBD-2 in oral homeostasis.

## Figures and Tables

**Table 1:** Basic demographic and clinical characteristics of the study participants.

Participants	*n*	Age (Years) (Mean ± SD)	Gender (*n*; %)	dt/DT = 0 (*n*; Mean ± SD)	dt/DT ≥ 1 (*n*; Mean ± SD)
Primary dentition	75	3.44 ± 0.43	Male (42; 56%)	30; 0.00 ± 0.00	45; 7.31 ± 4.82
			Female (33; 44%)		
Permanent dentition	78	12.63 ± 0.65	Male (34; 44%)	30; 0.00 ± 0.00	48; 6.19 ± 2.85
			Female (44; 56%)		

*n*—number; SD—standard deviation, dt/DT—decayed teeth.

**Table 2:** Mean salivary values of hBD-2 peptide and saliva pH in the study participants.

hBD-2 (ng/mL)	Mean ± SD	*p*-Value
Primary dentition	2.26 ± 1.48 2.42 (0.80–3.46) ^3^	0.044 ^1^
Permanent dentition	2.78 ± 1.08 2.69 (2.12–3.30) ^3^	
**Salivary pH**		
Primary dentition	7.32 ± 0.30 7.29 (7.06–7.54) ^3^	<0.001 ^2^
Permanent dentition	7.03 ± 0.31 7.09 (6.87–7.22) ^3^	

^1^ Mann–Whitney test; ^2^ *t*-test, SD—standard deviation; ^3^ median (interquartile range).

**Table 3:** Salivary parameters regarding dental caries in study participants.

hBD-2 (ng/mL)	Caries Free (Mean ± SD)	With Caries (Mean ± SD)	*p*-Value
Primary dentition	2.14 ± 1.19	2.51 ± 1.72	0.535 ^1^
Permanent dentition	2.60 ± 0.98	2.92 ± 1.12	0.150 ^1^
**Salivary pH**			
Primary dentition	7.34 ± 0.27	7.30 ± 0.31	0.595 ^2^
Permanent dentition	7.08 ± 0.36	7.00 ± 0.36	0.271 ^2^

^1^ Mann–Whitney test; ^2^ *t*-test, mean ± standard deviation.

**Table 4:** Spearman’s correlation analysis of salivary hBD-2 peptide concentration, unstimulated saliva pH, and dental caries in study participants.

		Primary Dentition		Permanent Dentition	
		Saliva pH	Ʃdt/DT	Saliva pH	Ʃdt/DT
**hBD-2**	*r_s_*	−0.067	0.076	−0.230	0.139
	*p*	0.566	0.515	0.043 *	0.224
	*n*	75	75	78	75
	95%CI	−0.298–0.164	−0.150–0.288	−0.430–−0.009	−0.102–0.360
	effect size	Negligible	Negligible	Small	Negligible
**Saliva pH**	*r_s_*	1	−0.180	1	−0.213
	*p*		0.121		0.061
	*n*		75		75
	95%CI		−0.381–0.037		−0.419–0.015
	effect size		Small		Small

*r_s_*—Spearman’s correlation coefficient; *p*-value; * significant for *p* < 0.05; *n*—number; 95%CI—95% confidence interval; dt/DT—decayed teeth.

## Data Availability

The data presented in this study are available from the corresponding author on request.

## Abbreviations

The following abbreviations are used in this manuscript:

hBD-2	Human β-defensin 2
WHO	World Health Organization
ELISA	Enzyme-linked immunosorbent assay
MAPK	Mitogen-activated protein kinases
NFκB	Nuclear factor kappa B
